# The Gloves-Bill: All-Gloves Versus No Gloves

**Published:** 1912-11-23

**Authors:** 


### PROBLEMS IN ADMINISTRATIVE MEDICINE.

(Carefully thought-out Contributions to this Section will be welcome.)

### The Gloves-Bill: All-Gloves versus No Gloves.

Since Semmelweiss. and Lister proved the immense value of cleanliness and the avoidance of pathogenic germs in surgical technique the details of operative treatment in a modern hospital have been almost revolutionised. This is a truism that needs no elaboration. Anyone acquainted with the history of modern surgery knows how successive changes have come about in the technique, and how we have progressed from the Listerian spray, which is now a museum curiosity, to present-day asepsis. The antiseptic procedure, briefly stated, was to render the pathogenic germs innocuous by robbing them of their vitality and minimising their chances of finding culture foci in the tissues of the patient. An elaborate ritual was performed by the surgeon, whose lustrations were sometimes made in five different fluids, all warranted to help in the process of devitalising the organisms. But it was soon found that solutions of chemicals strong enough to effect this purpose tended also to injure the wound tissue, to delay healing, and to introduce factors which were by no means desirable. At the same time rigid bacteriological investigation showed that the use of antiseptics did not afford absolute safeguards. In course of time the aseptic school gained in strength, and to-day in the best hospitals and institutions, notably those of Germany and America, asepsis has almost displaced antisepsis. Asepsis is not a modern innovation. Hippocrates counselled it, and Trotula di Ruggieri, the Salernitan doctress, boiled her instruments before she operated. But it is undoubtedly true that the practice of asepsis, so far as institutions are concerned, is a development of the last ten years, and, as such, hospital workers have to take it into account. We cannot here deal with the general views on the subject, nor with the question whether it is not better for the general practitioner, with limited means and armamentarium, to trust wholly to antiseptics than to attempt a necessarily imperfect aseptic technique. We wish merely to draw attention to one side of the matter, the special issue—namely, of the desirability that hospital surgeons should wear gloves when performing operations.

More than ten years ago the most perfect aseptic ritual was practised in the famous Breslau theatre, built by the surgeon Mickulitz from his own designs. English visitors who attended these operations were struck at once by the immense expenditure of trouble, labour, and money to obtain what were held to be ideal conditions, and by the relatively small percentage of perfectly aseptic wounds in the surgical wards. In the theatre all the workers were clothed in special dress; they wore gloves, goggles, and to the uninitiated looked like gollywogs. No instruments or dressings were touched by anyone except the surgeon and his immediate helper; everything was handed to them at the end of a long sterile forceps. The technique had a certain flavour of theatricality about it, and most of the ritual passed away when the great surgeon died, and his successor discarded all superfluities, contenting himself with simpler means. Indeed, the general tendency nowadays is to simplify the technique as much as possible. Operating theatres, which threatened at one time to raise the cost per bed in a new hospital by 7 per cent., are now built on lines of studied simplicity, for it has been acknowledged that perfect asepsis is an unattainable ideal, and that the best results can be obtained with much less expenditure of energy and money. These uniformly good results have been gained largely by the use of gloves. The modern surgeon is provided with thin rubber hand casings, which can be rendered absolutely sterile by boiling or dry heat. Armed with these he knows that, unless he errs in some gross way, he is perfectly safe in operating, provided always that he takes ordinary precautions to have his instruments and dressings sterile. From the administrative point of view, therefore, gloves are a necessity. Formerly they were made of thick rubber, and were consequently an expensive item; to-day they are thin and cheap, but even more efficacious since they allow of greater flexibility and delicacy in manipulation.

Yet it must be admitted that there are some well-known surgeons who do not habitually employ these safeguards, and who yet manage to show a percentage of septic cases as small as, or smaller than, their colleagues who invariably employ gloves. Trusting entirely to the old Listerian methods of antisepsis, these surgeons maintain that their technique is as successful as that of the more thoroughly aseptic operators. The *posse* of American surgeons who recently visited the London hospitals have put on record their astonishment at the excellent results obtained in one large theatre where the older system is followed by one of our most expert metropolitan surgeons. The objections to the habitual use of gloves in all cases are mainly these: the glove necessarily limits to a certain extent the finer movements of the fingers; it reduces tactile sensibility so that the operator has to train himself anew by the mere touch to recognise organs which he cannot see; it is a danger in so far that its use tends to make the operator careless or indifferent in thoroughly cleansing his hands preparatory to drawing on the gloves, and doubly so because the glove is liable to be pricked by a sharp instrument and to allow bacteria from the skin of the wearer to emerge through the opening and so contaminate the wound; and, finally, that the glove itself is difficult to sterilise.

All these objections are well founded, but they fall to the ground when the surgeon who employs gloves is a careful and methodical operator, who takes ordinary precautions to minimise all risks of sepsis. It is, no doubt, ludicrous enough to see an operating surgeon preparing for his case carefully insinuating his hand into the sterile glove and then drawing the glove firmly on by using his ungloved and not perfectly sterile hand; in this way the outside of the glove gets soiled, and, although consequent immersion of the gloved hands in sterile solutions may cleanse it again, the practice is not to be recommended. A greater danger lurks in the unconscious pricking of the glove by means of a needle or sharp pointed scalpel, or in the use of mended gloves, which can never be relied upon. In some institutions thin cotton gloves are used which can be effectively sterilised by boiling. Professor Spitzy, whose pioneering work on nerve suturing is well known, uses such gloves, and the fact that his technique is so uniformly successful and that his percentage of septic cases is remarkably low speaks well for his methods. Cotton gloves, however, still further impair tactile sensibility and easily get saturated with blood, so that several pairs must be used in the course of even a minor operation.

When we compare the results of surgeons who always wear gloves with those who never employ them there can be no question that the former have a lower percentage of septic cases than the latter. The exceptions are, of course, when the non-glove operator is exceedingly careful and takes the greatest pains to cleanse his hands thoroughly. Absolute data with regard to the percentage of septic wounds in clean cases are difficult to obtain, mainly because the subsequent dressing of the wounds, the nursing, and the state of the ward may greatly influence such percentages. Doubtless a clean and careful surgeon, who can rely upon his ward, his dressers, and his nurses, is justified in discarding gloves, especially if he is an expert and quick operator, in clean cases. That gloves are a necessity in septic operations is now generally recognised, since they protect the surgeon as well as the patient, and also serve as a protection to patients subsequently to be operated upon. But for the sake of uniformity alone, if for nothing else, the general use of gloves by operators should be insisted upon in every hospital. In private practice, too, the surgeon who wears gloves (provided always that he does not neglect other precautionary measures) is, from a medico-legal point of view, better protected in case something goes wrong than if he had disdained to wear gloves. The public has a right to demand that the surgeon should fall into line with his colleagues where a principle of protective cleanliness in technique is concerned. Surgeons on the staffs of London and other hospitals who have not been in the habit of wearing gloves, and who are confident that their percentage of septic wounds is as low as that of their glove-wearing colleagues, have loyally taken to the new *régime* and donned gloves for the sake of uniformity. Superintendents and secretaries will, therefore, find that the annual bill for gloves is an increasing one, and would do well to lay in a fairly large supply of stock sizes, since when bought in quantity these articles are comparatively cheap. In the German hospitals the tender price per pair has steadily declined; a few years ago it was half-a-crown; to-day excellent gloves are supplied at one shilling to one and sixpence per pair. Operating gloves are different from protective gloves used in the post-mortem room and in the septic wards; the latter are thicker, coarser, and generally more expensive.

One of the greatest objections to the use of gloves is undoubtedly the difficulty of sterilising them. Boiling spoils them, and dry heat is generally preferable. Each institution has its own methods for gloves and ligatures, the most common being simple boiling in a soda solution and rinsing in sterile salt solution afterwards. Where the iodine method is used in preparing the site of operation, and the surgeon and his assistants take care to cleanse their hands thoroughly before putting on the gloves, all concerned may rely that everything has been done to make the conditions of the operation as good as they can be when such gloves are worn by all who touch patient, dressings, or instruments.

